# How did the ‘state of emergency’ declaration in Japan due to the COVID-19 pandemic affect the acoustic environment in a rather quiet residential area?

**DOI:** 10.14324/111.444/ucloe.000009

**Published:** 2020-08-12

**Authors:** Kimihiro Sakagami

**Affiliations:** 1 Environmental Acoustics Laboratory, Department of Architecture, Graduate School of Engineering, Kobe University, Rokko, Nada, Kobe 657-8501, Japan

**Keywords:** acoustic environment, noise level, residential area, state of emergency in Japan, lockdown, COVID-19 pandemic, built environment, urban studies

## Abstract

The COVID-19 pandemic caused lockdowns in many countries worldwide. Acousticians have made surveys to monitor how cities became quieter under the lockdown, mainly in central areas in cities. However, there have been few studies on the changes in the acoustic environment due to the pandemic in the usually quieter residential areas. It may be expected to be different from the effect in ‘originally noisy’ areas. Also, the effect could be different in Japan, because the ‘state of emergency’ declaration there was different to lockdowns elsewhere. Considering these circumstances, this article reports the results of noise monitoring and makes some observations on the acoustic environment in residential areas far from city centres, to provide an example of how the acoustic environment was affected by the state of emergency declaration due to the COVID-19 pandemic in Japan. The results showed that the reduction of noise levels was somewhat less than that reported in large cities. Also, comparing the results after the cancellation of the state of emergency, the noise level increased again. However, observations of noise sources imply that a possible change in human behaviour may have also affected the acoustic environment.

## Introduction

On 16 January 2020, the first Japanese person infected with coronavirus (COVID-19) was discovered and the case was reported in newspapers a few days later [1]. Thereafter, the Government of Japan announced the introduction of precautionary measures. However, the number of infected cases continued to rise, and finally, on 7 April, the government declared a ‘state of emergency’ in seven prefectures. On 16 April it was expanded to all prefectures, and it continued until 25 May 2020, when the declaration was formally cancelled for all prefectures.

A state of emergency is not the same as a lockdown: under a state of emergency, the national and local government can ‘request’ or ‘instruct’ the population not to engage in particular activities which could possibly spread an infection, for example, eating amenities had to shorten their opening hours or close until the declaration was cancelled [2]. Therefore, although people were requested to stay home unless they had essential needs or were essential workers, they could, taking careful precautions, go out to work or go shopping.

In fact, many people worked from home, whereas other workers commuted by train, bus, or using other public transport. Schools at all levels were closed and most universities switched to online classes. Therefore, it is indeed the case that, during this period, there were far fewer people in the centre of the country’s cities, although some human activity was observed.

In short, a state of emergency is not as strict as a lockdown, as occurred in many other countries. However, it was reported that the behaviour of people changed after its cancellation [3].

Discussions on various aspects are needed to consider urban environments in light of this pandemic [4,5] here, this paper focuses on the urban acoustic environment. There are already many acousticians who have reported changes in the acoustic environment, particularly noise levels, in the central areas of various cities [6–9].

However, in this paper, some observations on the acoustic environment in a rather quiet residential area are reported, for the following reasons: (1) Most reports concentrate on urban areas that originally had higher noise levels and discuss how noise levels were reduced, but it seems that there are fewer reports of observations in formerly quiet residential areas. Given the fact that a considerably swathe of a city can be categorised as residential, it is important to know what the change was like in such an area. (2) As already mentioned, Japan’s state of emergency was not exactly the same as the lockdown that occurred in other countries, which means the activities and behaviours of people may have been different from those in the countries under lockdown in the strict sense.

Regarding acoustic environment (and soundscape) research, Nagahata [10] published an archive of sound data recorded in Fukushima, Sendai and the greater Tokyo area. This archive is invaluable for further analyses, including perceptual evaluation. In this paper, only measured noise levels are presented with some noticeable perceptual features heard during the measurement. The survey was carried out from 13 to 28 May 2020, to compare the results during the state of emergency and after it was cancelled. Also, for reference, the author’s data were compared with measurements in November and December 2019 in the same area. The survey’s main purpose is to provide an example of the extent to which the acoustic environment changed during the state of emergency in a usually quiet residential area, and is not intended to be generalised, as this is a case study in one limited area.

## Surveyed area and method

**Figure 1:**
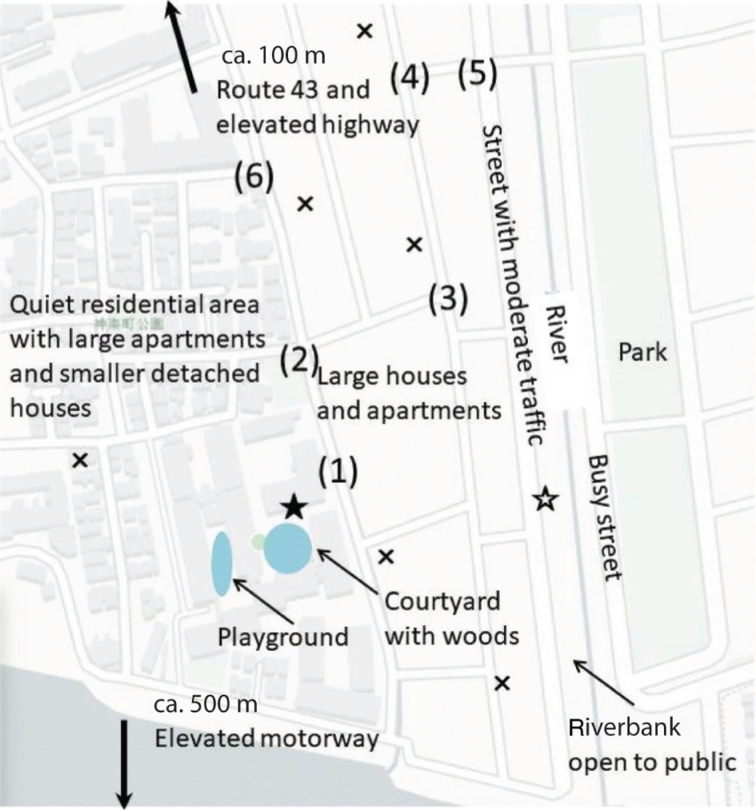
Map of the surveyed area (eastern edge of Kobe city). The numbers (1) to (6) show measuring points. The black star is the measuring point for fixed-point observation. The white star is the measuring point for monitoring noise levels in the public space (river). The Xs show construction sites.

Figure 1 is a map of the surveyed area, the eastern edge of the city of Kobe. All measuring points are marked in the figure. Construction sites are marked with ‘X’. At these points, construction work was stopped from the end of February 2020, but in most of the sites, work resumed in early May 2020. The author does not have knowledge of the circumstances behind this fact.

This area is designated as scenic (construction of buildings taller than five storeys is prohibited) and residential (no commercial buildings are allowed). This area is occupied mainly by two-storey detached houses; however, there is also a somewhat large number of small and large apartment buildings of three to five storeys. In 2019, the noise levels of this area were surveyed as a follow-up to the author’s previous study [11], and the results demonstrated that it was rather quiet except for the occasional sound of passing cars. For other datasets in the same area, three sets of geo-referenced sound data taken in August–October 2019 using an iPhone XS and three apps (dB Meter Pro, Decibel X and SoundLog) are available in reference [11] in which instantaneous sound pressure level (SPL) (dBA, slow, peak) taken using a dB Meter Pro and Decibel X and L_Aeq_ taken using SoundLog can be found.

There is a river in the east of this area, the riverbed of which is wide and accessible to the public, and along the riverbank there is a rather busy street with moderate traffic volume. In the north is Route 43 and an elevated motorway, both of which are relatively busy, and in the far south there is another highway. From those main sources of traffic, continuous noise can be heard, albeit faintly, throughout the day.

Measurements of equivalent continuous A-weighted sound pressure level, L_Aeq_ (T = 30 s) were performed using the NoiseCapture app for Android [12], which is widely known and used as a reliable tool for monitoring noise, although it is not as good as a professional Class 1 sound level meter (SLM). The device used was an Android tablet (Teclast tPad P80X). Although a detailed accuracy check was not performed, it was decided to use the NoiseCapture app (for Android only) for this work, as it has the advantage of being able to record both GPS data of measured points and perceived noise source. The app on this device was calibrated using a Class 1 SLM before the laboratory closed due to the state of emergency.

The measurement accuracy was checked later in comparison with a Class 1 SLM after the survey in a laboratory test, and it was confirmed that this measurement system presents reasonable accuracy for the present purpose (see Appendix A). Also, the short duration of the average (T = 30 s) was taken because the sounds measured in this environment were almost at steady state. Of course, the results obtained by this short duration will not be suitable to compare with the data obtained by other authors, for example, Asensio et al. [13], however, it is considered useful for the purpose of this work, which is limited to understanding the acoustic environment of this particular area.

## Results and observations

### The noise levels at points (1) to (6)

Table 1 shows the results of the noise levels (L_Aeq_, T = 30 s) measured at points (1) to (6). Averaging over a rather short time (T = 30 s) was chosen as the noise in this area showed little time fluctuation and had almost steady state noise. The measurement was repeated once at each point at the same hour (10:00–11:00). Levels larger than 60 dBA were likely to be affected by nearby construction sites and/or increased traffic volume. The levels in data taken during the state of emergency (13 May 2020) were higher than those in 2019: this is likely due to the increased traffic and/or construction works. The same observation can be applied to those taken after the state of emergency was cancelled (25 May 2020). Removing the data that may be affected by construction noise, the three sets of data show some difference, but it is not very significant. For example, when the levels larger than 60 dBA were removed from all the datasets, the average levels were 50.5, 51.6 and 52.9 dBA. The levels relative to the average value of the 2019 survey are also shown in Table 1. This may be interpreted as that both during the state of emergency and after its cancellation, the noise level was tending to increase. Thus, a possible effect can be observed and interpreted as the increased traffic volume observed and construction works in this area in May 2020: During the state of emergency, a certain number of people used either a car or a motorbike for commuting, shopping and travelling for various purposes, which would have increased traffic volume in this area.

**Table 1.** Noise levels (L_Aeq_), dBA, at points (1) to (6).

Point	Nov and Dec 2019	13 May 2020	25 May 2020
1	52.3	45.7	44.7
2	48.6	63.7	50.1
3	53.9	64.6	61.1
4	48.5	59.3	56.9
5	65.1	55.4	59.4
6	49.3	45.9	53.5
Average 1–6	52.9	55.8	54.3
Relative level (to 2019)	0	+2.9	+1.4

For reference, a previous survey in the same area [except for point (6)] in August–October 2019 [11] showed that: instantaneous noise levels (dBA, slow, peak) were 44.8 dBA [minimum, point (1)] to 64.8 dBA [maximum, point (5)]. This range roughly corresponds to the given results, where it can be inferred that, although the variation due to the day is observed in Table 1, the noise levels fall into the range observed in those observed in the usual state.

Although the average values seem to suggest a slightly increasing trend of the noise levels in this area during and after the state of emergency, and by observation this could be attributed to increased traffic volume and construction noises, it was not conclusive enough as the number of measurements could not be enough and the dataset was not complete.

### Fixed-point observations

#### Noise levels

The L_Aeq_ on the balcony of an apartment (third floor of a five-storey building) was monitored from 13 to 19 May 2020 (during the state of emergency) and from 23 to 28 May 2020 (after the cancellation). The measurement was performed every hour from 07:00 to 19:00, and at 24:00 for reference. The measurement point faced a courtyard with many trees, and it was surrounded by four premises of five-storey apartments. Therefore, this point is usually very quiet (around 40 dBA at midnight). There are occasional children’s activities in the courtyard during the daytime. Also, there is a playground for children outside the apartment complex which is sometimes very vibrant in the daytime through to the early evening.

The measured values were expected to show some consistent trend; however, there was no such trend, and they vary randomly from day to day. However, Saturday and Sunday show different trends from each other, and are different from weekdays. Therefore, the data for weekdays were averaged, and data for Saturday and Sunday are presented separately.

**Figure 2:**
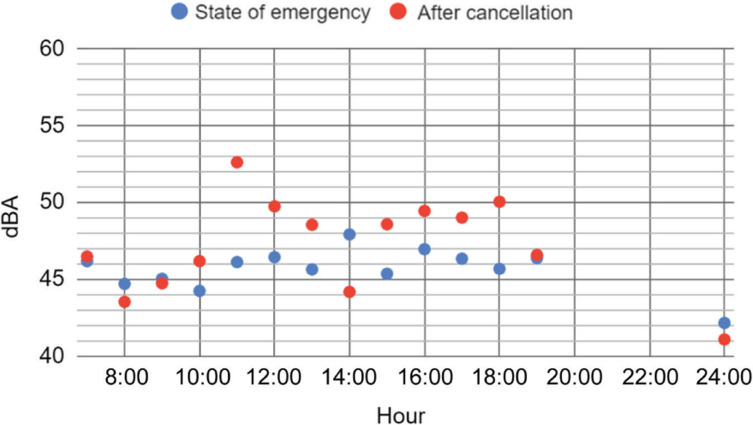
Comparison of the day history of the noise levels (L_Aeq_, T = 30 s) of weekdays during the state of emergency (blue) and after its cancellation (red). The plot of each hour is the averaged value over all weekdays during the observation period.

Figure 2 compares the noise levels, L_Aeq_, the duration was again taken as T = 30 s as the noise showed only small time fluctuations (in some cases 10 s to avoid sudden loud sounds) for weekdays (averaged for each hour) measured during the state of emergency and after its cancellation. As was observed, during the state of emergency, noise levels did not show significant variation in a day and were almost constant, therefore there is no strong correlation (the correlation coefficient was *R* = 0.52); however, after the cancellation, the levels increased somewhat (the average difference is 2.5 dBA) except for early morning and at midnight. This difference is considered as significant (*p* < 0.05, *t* = 2.55). The large difference observed at 11:00 was considered very likely to come from construction noise at neighbouring sites.

**Figure 3:**
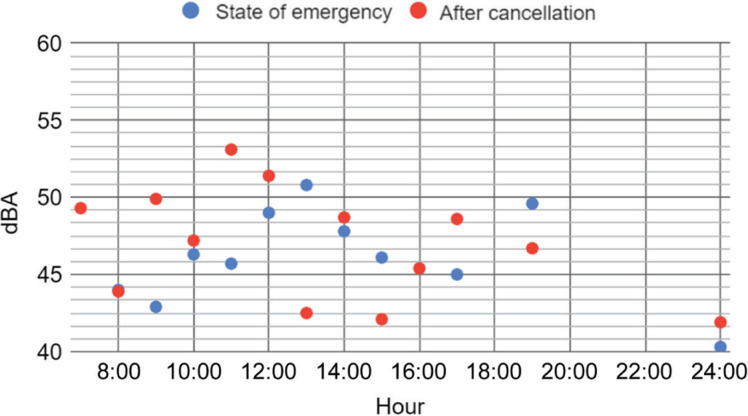
Comparison of the day history of the noise levels (L_Aeq_, T = 30 s) of Saturday during the state of emergency (16 May 2020) (blue) and after its cancellation (23 May 2020) (red).

**Figure 4:**
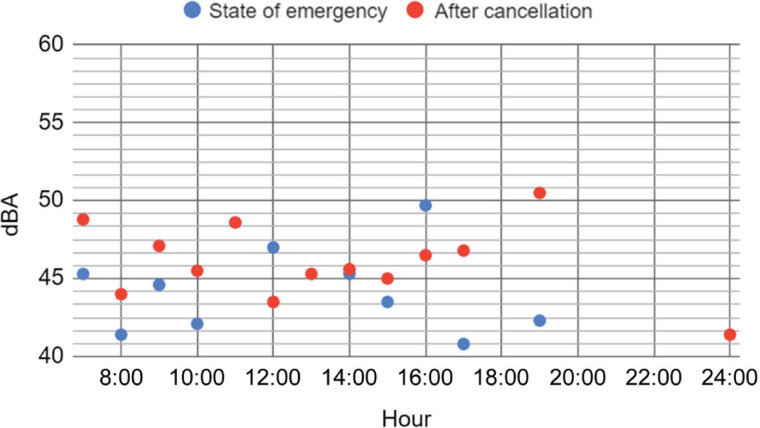
Comparison of the day history of the noise levels (L_Aeq_, T = 30 s) of Sunday during the state of emergency (17 May 2020) (blue) and after its cancellation (24 May 2020) (red).

Figures 3 and 4 show the measured values for Saturday and Sunday, respectively. Results of analyses of variance show that the difference between during and after the state of emergency was not significant for Saturday (*p* = 0.55), but was somewhat significant for Sunday (*p* = 0.1). Although one set each was given, the noise level on Sunday after the cancellation of the declaration (average 46 dBA) may have been larger than during the state of emergency (average 44 dBA). Although it is hard to find a consistent tendency, Saturday can be considered to be noisier than Sunday (not statistically significant: *p* > 0.1). In particular, from observations it was understood that, after the cancellation of the state of emergency, residents tended to go out in the morning. In many cases, people generally tended to go out on Saturday and stay home on Sunday to recharge for work on Monday. This behaviour was reflected in these figures.

Putting inferences aside, considering these three graphs, we obtain only a few observations: (1) During the state of emergency this area was slightly quieter, but no consistent tendency of day-to-day noise-level variation was shown in the results; (2) Saturday was assumed to be the noisiest day of the week in both cases.

### Perceived noise sources

**Figure 5:**
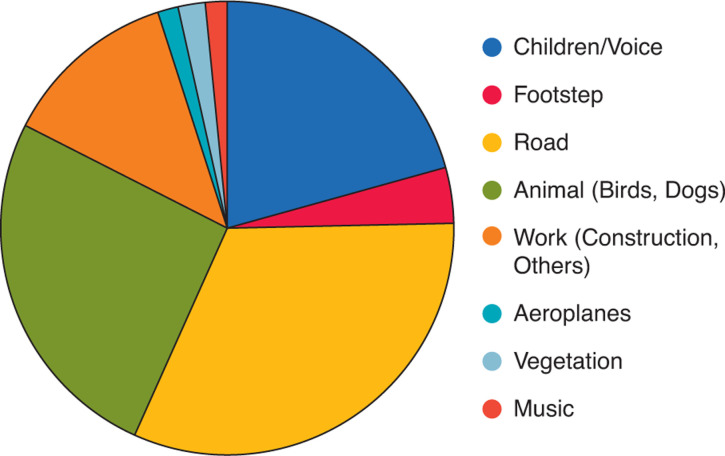
Main noise sources perceived during the measurements under the state of emergency.

**Figure 6:**
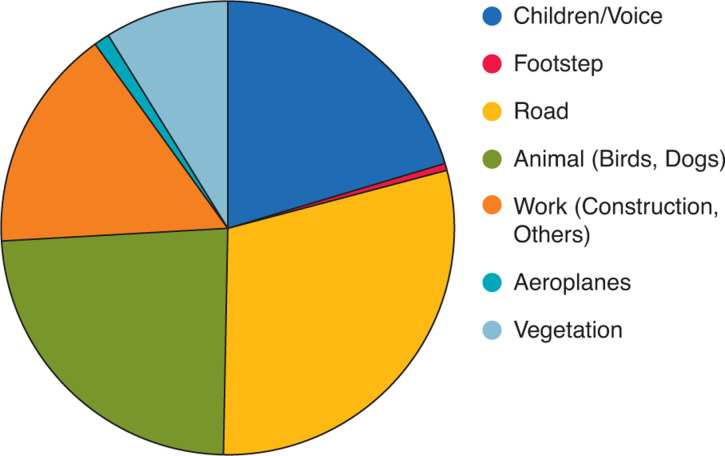
Main noise sources perceived during the measurements after the state of emergency was cancelled.

Regarding the noise sources, perceived noise sources are shown according to their percentage in Fig. 5 (during the state of emergency) and Fig. 6 (after its cancellation).

**Figure 7:**
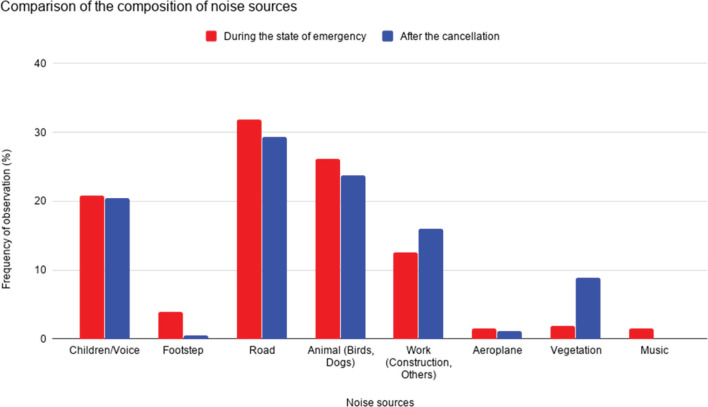
Comparison of the compositions for noise sources of weekdays and weekends (Figs 5 and 6). Note that the percentage of observation of music after the cancellation of the state of emergency was zero.

Comparing these two graphs, and also from Fig. 7, in which two results are compared, it can be seen that the main noise sources are not different from each other. In this area, as mentioned in the section Surveyed area and method, the road traffic noise from the two main traffic sources (31.9% during the state of emergency and 29.3% after its cancellation) is noticeable throughout the day, although it is not very strong; therefore, it was perceived in most measurements. Voices and children are the main sources in both cases (under the declaration 20.8%, after the cancellation 20.4%). After the cancellation of the state of emergency, all schools remained closed and the children stayed home, which is one of the reasons why their voices were a main source of noise. By perceptive observation, it was noted that children’s voices were most dominant in the morning and early evening. This is assumed to be a result of their daily behaviour, which may change after school resumes.

As for the contribution of animals (under the declaration 26.1%, after the cancellation 23.8%), which in this area mainly pertained to birds, it was more frequently and loudly perceived during the state of emergency. This may have been due to the variation of the noise levels from other sources, or possibly due to the relationship between the behaviours of birds and humans – as mentioned below, the river and park where birds had often been seen before were rather crowded during the state of emergency with people who may have finished for the day working from home and children who were not going to school; this made these places somewhat noisier, which could drive birds from the river and the park.

As for some of the sources that made a small contribution, for example, music was sometimes heard during the state of emergency, however, it was not heard after its cancellation. Regarding music, the most often heard was recorders, probably played by primary school pupils. As regards aeroplanes, the contribution was low at both times as flights were reduced both during and after the declaration of the state of emergency.

As such, regarding the source of noise observed, the main sources were not significantly changed during the state of emergency and after its cancellation: almost the same sounds were heard during both periods.

### A small observation at the riverside

As mentioned already, the river and its banks were rather more crowded than before, with people taking walks for pleasure especially in the early evening, perhaps after a day’s work. There were children around during daytime and early evening, and this could also be one of the factors increasing the noise levels in the surrounding area.

Table 2 shows the results of the measured noise levels (L_Aeq_, T = 30 s) at the point marked with the white star on the map in Fig. 1.

**Table 2.** Noise levels at the fixed point on the riverside, L_Aeq_, T = 30 s, dBA.

Hour	15:30	17:30
24 May 2020	50.5	51.6

Although only a 1 dBA change was observed, early evening showed a higher level than mid-afternoon, which is in agreement with the perceived observations.

**Figure 8:**
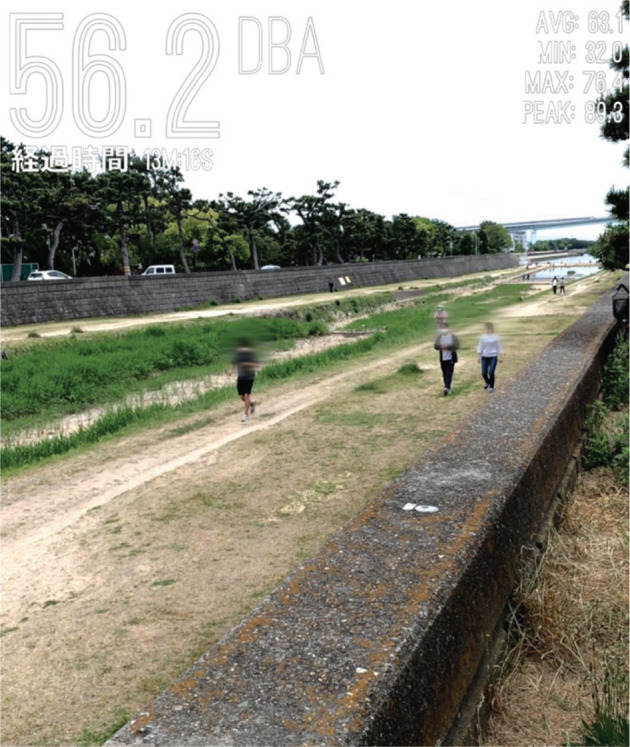
An example of a photograph overlaid with measured noise level (dBA, slow, peak) showing activities of people on the riverbanks. This photograph was taken at the same point as that of measurements shown in Table 2.

Just as an example, a photograph taken at the same point is shown in Fig. 8 (at 13:57 on Saturday, 9 May 2020, during the state of emergency). This was taken using the Decibel X Pro app on an iPhone XS so that a measured value of instantaneous noise level, dBA (slow, peak), was overlaid. The app on the device was calibrated with a Class 1 SLM before the closure of the laboratory due to the pandemic. Although it is a rather extreme example, it describes people’s activities on the riverbed, and the noise level was not as low as it would be usually.

## Conclusions

In this paper, some observations were made on the effect of the state of emergency due to the COVID-19 pandemic in Japan on the acoustic environment in a rather quiet residential area. Recent reports suggest that the effect of lockdown reached several dBA in most cases. However, in the area observed in the present survey, the effect was ca. 1–2 dBA, that is, during the state of emergency it was only slightly quieter. This effect was much smaller than the drastic reduction reported from busy areas such as city centres, etc. [14]. This can be interpreted as the difference between a lockdown and a state of emergency, and the behaviour of people resulting from that difference. Also, a large effect may not be possible in an usually quiet area.

For reference, the author performed a small survey asking 12 students about their impression on the change in the acoustic environment during the state of emergency. A few students who live in quiet areas stated there had been no significant change. However, some students claimed that it had gotten quieter, and they became aware of neighbourhood noise such as voices, TV sounds, music, and other daily noises. Some of them claimed that it was annoying, and became rather sensitive even after the cancellation of the declaration and the acoustic environment returned to normal as before the state of emergency was declared.

Although in this work professional precision instruments were not available due to the restricted circumstances, moreover the work was focused on just one particular area, the present results and discussions are very limited. Therefore, the generalisation or the ability to derive universal conclusion of these results were not intended. However, the results and observations in this work could possibly give some insights into the relationship between human behaviour and the acoustic environment. Also, the author expects that a follow-up survey may give some more insight as the situations around this area are ever changing.

## Figures and Tables

**Figure A1:**
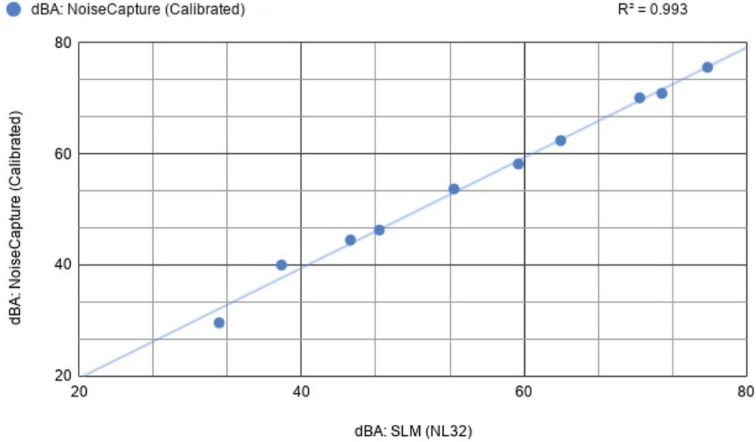
Results of the accuracy check of NoiseCapture. The vertical axis shows the values measured by NoiseCapture, and the horizontal axis shows those measured by a Class 1 SLM (RION, NL-32). The line shows linear regression (R^2^ = 0.993).

## Data Availability

The datasets generated during and/or analysed during the current study are available from the corresponding author on reasonable request.

## Article note

Regarding the possible effect of the schools restarting mentioned in section **Perceived noise sources** [on 1 June when all schools (except for universities) resumed], however, at the time of writing, school hours vary from school to school depending on the grade, academic level and school policies. On this point, a follow-up survey would be of some interest to observe any possible gradual change in the acoustic environment.

## Appendix A: Accuracy check of NoiseCapture on the Android tablet (P80X) used

After the survey, a simple check of the accuracy of the measured values by the system used in this work was carried out. Keeping the app and devices in the same setting and condition as was used in the measurement, a simple check was performed according to the following procedures [11]: pink noise was emitted from a 10-cm full-range loudspeaker enclosed in a bass-ref cabinet. The noises emitted at various levels were measured by both NoiseCapture on the tablet and a Class 1 SLM (RION, NL-32) simultaneously. The measured values by both the system were checked to see how much they were in agreement. The results are plotted in Fig. A1.

As is observed, the measured values show reasonably good agreement with those by a Class 1 SLM. The agreement deteriorated below 40 dBA; however, the discrepancies were within 3 dBA, which is sufficient for the present purpose in which the relative values are important and most measured values were above 40 dBA. The line in the figure is a linear regression line which almost coincides with diagonal line, and R^2^ = 0.993, which is regarded as sufficiently high in the light of the present purpose.
